# Isolation and Characterization of Yeasts from Rumen Fluids for Potential Use as Additives in Ruminant Feeding

**DOI:** 10.3390/vetsci8030052

**Published:** 2021-03-19

**Authors:** Chanon Suntara, Anusorn Cherdthong, Metha Wanapat, Suthipong Uriyapongson, Vichai Leelavatcharamas, Jutaporn Sawaengkaew, Pin Chanjula, Suban Foiklang

**Affiliations:** 1 Tropical Feed Resources Research and Development Center (TROFREC), Department of Animal Science, Faculty of Agriculture, Khon Kaen University, Khon Kaen 40002, Thailand; Chanon_su@kkumail.com (C.S.); metha@kku.ac.th (M.W.); suthipng@kku.ac.th (S.U.); 2 Fermentation Research Center for Value Added Agricultural Products (FerVAAP), Department of Biotechnology, Faculty of Technology, Khon Kaen University, Khon Kaen 40002, Thailand; viclee@kku.ac.th; 3 Department of Microbiology, Faculty of Science, Khon Kaen University, Khon Kaen 40002, Thailand; sjutap@kku.ac.th; 4 Animal Production Innovation and Management Division, Faculty of Natural Resources, Hat Yai Campus, Prince of Songkla University, Songkhla 90112, Thailand; pin.c@psu.ac.th; 5 Faculty of Animal Science and Technology, Maejo University, Chiangmai 50290, Thailand; bungung@hotmail.com

**Keywords:** ruminal yeast, screening, isolation, biomass of yeast, cellulase enzyme

## Abstract

*Saccharomyces cerevisiae* is a yeast strain often used to improve the feed quality of ruminants. However, *S. cerevisiae* has limited capacity to provide biomass when inoculated with carbon sources and a low ability to produce cellulase enzymes. Here, we hypothesized that yeast in the rumen produces a large amount of biomass and could release cellulase enzymes to break down fiber content. Therefore, the aim of this study was to screen, isolate and identify yeast from the rumen fluids of Holstein Friesian steers and measure the efficiency of biomass production and cellulase activity. A fermentation medium containing sugarcane molasses as a carbon source and urea as a nitrogen source was optimized. Two fistulated–crossbred Holstein Friesian steers averaging 350 ± 20 kg body weight were used to screen and isolate the ruminal yeast. Two experiments were designed: First, a 12 × 3 × 3 factorial was used in a completely randomized design to determine biomass and carboxymethyl cellulase activity. Factor A was the isolated yeast and *S. cerevisiae*. Factor B was sugarcane molasses (M) concentration. Factor C was urea (U) concentration. In the second experiment, potential yeasts were selected, identified, and analyzed for 7 × 4 factorial use in a completely randomized design. Factor A was the incubation times. Factor B was the isolated yeast strains, including codes H-Khon Kaen University (KKU) 20 (as *P. kudriavzevii*-KKU20), I-KKU20 (*C. tropicalis*-KKU20), and C-KKU20 (as *Galactomyces* sp.-KKU20). Isolation was imposed under aerobic conditions, resulting in a total of 11 different colonies. Two appearances of colonies including asymmetric colonies of isolated yeast (indicated as A, B, C, E, and J) and ovoid colonies (coded as D, F, G, H, I, and K) were noted. Isolated yeast from the rumen capable of providing a high amount of biomass when inoculant consisted of the molasses 15% + urea 3% (M15 + U3), molasses 25% + urea 1% (M25 + U1), molasses 25% + urea 3% (M25 + U3), and molasses 25% + urea 5% (M25 + U5) when compared to the other media solution (*p* < 0.01). In addition, 11 isolated biomass-producing yeasts were found in the media solution of M25 + U1. There were 4 isolates cellulase producing yeasts discovered in the media solution of M25 + U1 and M25 + U5 whereas molasses 5% + urea 1% (M5 + U1), molasses 5% + urea 3% (M5 + U3), molasses 5% + urea 5% (M5 + U5), molasses 15% + urea 1% (M15 + U1), molasses 15% + urea 3% (M5 + U3), and M25 + U3 were found with 2, 3, 1, 2, 1, and 2 isolates, respectively. Ruminal yeast strains H-KKU20, I-KKU20, and C-KKU20 were selected for their ability to produce biomass. Identification of isolates H-KKU20 and I-KKU20 revealed that those isolates belonged to *Pichia kudriavzevii*-KKU20 and *Candida tropicalis*-KKU20 while C-KKU20 was identified as *Galactomyces* sp.-KKU20. Two strains provided maximum cell growth: *P. kudriavzevii*-KKU20 (9.78 and 10.02 Log cell/mL) and *C. tropicalis*-KKU20 (9.53 and 9.6 Log cells/mL) at 60 and 72 h of incubation time, respectively. The highest ethanol production was observed in *S. cerevisiae* at 76.4, 77.8, 78.5, and 78.6 g/L at 36, 48, 60, and 72 h of incubation time, respectively (*p* < 0.01). The *P. kudriavzevii*-KKU20 yielded the least reducing sugar at about 30.6 and 29.8 g/L at 60 and 72 h of incubation time, respectively. The screening and isolation of yeasts from rumen fluids resulted in 11 different yeasts being obtained. The potential yeasts discovered in the rumen fluid of cattle were *Pichia kudriavzevii*-KKU20, *Candida tropicalis*-KKU20, and *Galactomyces* sp.-KKU20. *P. kudriavzevii*-KKU20 had higher results than the other yeasts in terms of biomass production, cellulase enzyme activity, and cell number.

## 1. Introduction

The use of microbial additives such as yeast cultures has recently become common practice in ruminant nutrition [1]. In addition, the introduction of yeasts in the ruminant diet could replace antibiotic use as a growth promoter [2]. Yeast can act as a growth promoter by manipulating the ruminal environment so that it becomes more suitable for microorganisms. Yeast’s important roles include oxygen scavenging by reducing the amount of oxygen in the rumen and increasing the survival of other anaerobic microorganisms supplying essential nutrients such as mannooligo saccharide, protein or protein amino acid chelates, vitamins (B-complex), and minerals to other microorganisms upon cell lysis in the rumen; yeast can also stimulate the immune system in the hindgut [3]. These mechanisms affect animals by leading to maximum microbial population in the rumen, which increases their effectiveness in terms of their subsequent productivity [4]. Previous studies have confirmed that supplementation of *S. cerevisiae* as a direct feed can improve animal performance [5]. Other strategies apply *S. cerevisiae* and enhance feed quality as a feedstuff or as a byproduct by multiplying the yeast in the media solution; here, molasses (M) is the carbon source and urea (U) is the nitrogen source. This can expand the yeast population before treatment [6]. Previous experiments showed that the byproduct can be fermented with yeast for positive effect; its benefits are approximately the same when directly fed to animal [7].

The application of *S. cerevisiae* to fermented durian hull and ethanol waste (from cassava pulp) could enhance rumen fermentation, increase the ruminal bacteria population, and the average daily gain (ADG) of crossbred Brahman-Thai native cattle [8,9]. In addition, *S. cerevisiae* (plus malic acid) fermented with cassava pulp was suggested as a 50% replacement for soybean meal (SBM) in a concentrate diet for crossbred Brahman-Thai native beef cattle [10]. This achievement is attributable to the proliferation of the yeast cells and amino acids supply to the animal [9]. In addition, some studies have tried improving rice straw (RS) via fermentation with *S. cerevisiae*.

In Thailand, one of the most abundant by-products is rice straw (RS); it is regularly used as a roughage source for ruminant animals [11]. The nutritional values of RS include crude protein 3–4% and NDF 70.2%. The energy level from the total digestible nutrient (TDN) is considered low [12]. Although there are several approaches for improving the quality of RS, increasing protein and fiber digestion are the main pathways to success [12]. To date, there is still little available information about the use of *S. cerevisiae* to improve RS, and most of that research is not focused on RS fiber [13]. *S. cerevisiae* also may have a limited capacity to provide biomass when inoculated in high-glucose conditions before being used to treat byproducts especially RS.

This phenomenon is supported by Wardrop et al. [14], who stated that *S. cerevisiae* provides seven-fold less biomass than *Kluveromyces marxianus* when cultured in a media solution with high glucose. This is clearly true for *S. cerevisiae* due to the high ethanol yield produced. This is because glucose in *S. cerevisiae* could inhibit PDH and produce ethanol instead [15]. This phenomenon restricts the chances of animals receiving highly nutritious components from yeast cells, such as protein, essential amino acids, and vitamins. Therefore, using a different yeast strain may produce interesting and useful results.

The use of ruminal yeast as a feed additive has been studied [16], and yeasts isolated from the rumen appear to be much better for ruminant animals than those from other sources [17]. The capacity to develop biomass or other desirable properties, in particular the production of cellulase enzymes, has not yet been previously studied in rumen-isolated yeast. This is despite past studies suggesting that cellulase can be formed by some strains of yeast [18]. Our motivation here was to avoid the limitations of *S. cerevisiae* and determine the enzyme properties and thus enhance the value of RS.

Before the yeast inoculant can ferment with RS, we need to know the strains of yeast present in the rumen, the conditions for their growth, and their properties. A fermentation medium containing sugarcane molasses as a carbon source and urea as a nitrogen source was modified. We hypothesized that the yeast in the rumen produces a large amount of biomass and could release cellulase enzymes to break down the fiber content. Therefore, our goal was to screen, isolate, and identify yeast from the rumen fluids of Holstein Friesian steers and measure the efficiency of biomass production and cellulase activity.

## 2. Materials and Methods

All procedures involving animals in the metabolism studies were approved by the Institutional Animal Care and Use Committee of Khon Kaen University (KKU) (record no. IACUC-KKU 38/62).

### 2.1. Screening and Selection Potential Yeasts in the Rumen

#### 2.1.1. Animals, Diet and Isolation Procedure

The study was conducted at Tropical Feed Resources Research and Development Center (TROFREC), Department of Animal Science, Faculty of Agriculture, Khon Kaen University (KKU), Thailand. Two fistulated–crossbred Holstein Friesian steers, averaging 350 ± 20 kg body weight, were used as rumen inocula donors.

Two fistulated dairy steers were held in independent pens and individually fed ad libitum rice straw as roughage and 0.5% BW concentrate diets (16.0% crude protein (CP) and 75.0% total digestible nutrient (TDN) according to NRC [19]); they were given clean, fresh water and mineral blocks. The steers were fed at 07:00 a.m. and 04:00 p.m. The diet was calculated to meet animal requirements and with adequate nutrients for support microorganism in the rumen thus allowing this to occur for 7 days before the rumen fluid was obtained. According to Sirisan [20], the ruminal fluid was collected only once on day 7 from each fistulated steer via a rumen cannula 4 h after the morning feed. Rumen fluids from the two steers were mixed well before being filtered through a cheesecloth folded to form 4 layers. The fluid was filtered into a bottle and placed immediately into an ice bucket (4 °C). Samples were transported to the laboratory within 15 min. In the laboratory, 1 mL of ruminal fluid from each animal was diluted with 0.85% sodium chloride to 1:10, 1:100, and 1:1000 for a total plate count. Each ruminal fluid dilution was spread over a yeast–malt extract (YM) agar plate (HiMedia Laboratories Pvt. Ltd., India), which was then incubated at 39 °C for 72 h. The inoculant plate was dissolved in distilled water and sterilized for 15 min at 121 °C via autoclaving. The YM agar consisted of malt extract (3 g/L), yeast extract (3 g/L), peptone (5 g/L), agar (20 g/L), and glucose (10 g/L).

#### 2.1.2. Morphological Characterization

The streaking method was used to grow yeast colonies on agar media; samples were moved and regrown on another YM agar plate and then incubated for 7 days at room temperature. Yeast colonies were examined under a 40× light microscope. The isolated yeast strains were identified by studying different morphological characteristics [21]. The appearance of the yeast colonies was recorded: size, shape, convexity, surface, and color of colonies for purification. Colonies growing along the points of the streak were moved, purified, regrown in a YM broth (HiMedia Laboratories Pvt. Ltd., Mumbai, India), and kept in the refrigerator at 4 °C as stock colonies of yeasts. The isolates were then subjected to an initial assessment to determine their biomass and cellulase production ability by inoculating them in different solutions of sugarcane molasses, urea, and carboxymethyl cellulose (CMC).

#### 2.1.3. Determination of the Biomass and Carboxymethyl Cellulase Activity

##### Experimental Design and Preparation of Media Solution

The current study was conducted at the Fermentation Research Center for Value Added Agricultural Products (FerVAAP), Department of Biotechnology, Faculty of Technology, Khon Kaen University, Khon Kaen, Thailand, from June 2018 to September 2018.

To assess the growth of isolated yeasts in different conditions, we optimized a fermentation medium containing sugarcane molasses as a carbon source and urea as a nitrogen source to measure the efficiency of biomass production and cellulase activity. A completely randomized trial with a 12 × 3 × 3 factorial design was used, including 12 yeasts (11 isolated from the rumen and one control), 3 sugarcane concentrations (at 50, 100, and 250 g/L distilled water), and 3 urea concentrations (at 10, 30, and 50 g/L distilled water); two replicates were carried out for each treatment. Fermentation media were prepared by the addition of sugarcane molasses as a carbon source (Khon Kaen Dairy cooperative Co., Ltd., Khon Kaen, Thailand), urea as a nitrogen source (Saengtawee Panit Co., Ltd., Khon Kaen, Thailand), and carboxymethyl cellulose (CMC) 10 g/L distilled water as a stimulant substrate (Chemipan Co., Ltd., Bangkok, Thailand).

The media solution was autoclaved at 121 °C for 15 min to allow the media solution to cool down to room temperature before the addition of 70% H_2_SO_4_ to adjust the pH to 3.5 [10]. The pH levels of the fermentation media with isolated yeast and that of the sugarcane molasses with urea were measured by a glass electrode pH meter (Hanna Instruments, Inc., Woonsocket, RI, USA). The media were placed into 250 mL Erlenmeyer flasks. The flasks were filled to 100 mL, and 1 mL of isolated homogenous yeast suspension from the rumen (about 10^6^ cells per mL) was kept in aseptic conditions. Flasks were cotton plugged before incubation in an incubator–shaker machine [22].

Different levels of sugarcane molasses and urea were prepared with 100 mL of solution in a 250-mL Erlenmeyer flask by adding a single colony from a stock culture of all isolated and *S. cerevisiae* starter cultures (10^6^ cells) to the fermentation media. The cultures were then inoculated in an incubator–shaker at 30 °C and 150 rpm for 72 h. One milliliter of fluid culture was collected in a 1.5 mL tube with 2 replicates at each treatment condition to measure biomass and cellulase activity. After determination, the optimum conditions for yeast to produce the greatest amount of biomass and cellulase were recorded, and the highest yields of the yeast colonies were also selected for further experiments including cell counts, reducing sugar, and ethanol production.

Biomass was determined as described by Johnson et al. [23]. Here, 1 mL of cultured liquid sample was centrifuged in a 1.5 mL tube at room temperature at 10,000× *g* for 10 min. After centrifugation, the sample was separated into two parts: The precipitate parts or cell pellets were first washed with distilled water and then dried at 105 °C until the weight of biomass remained constant. Second, the crude supernatant was collected to measure the activity of carboxymethyl cellulase as determined using the DNS reagent via a colorimetric method according to Miller [14]: Here, 0.5 mL of crude supernatant was applied to 0.5 mL of 1% (*w*/*v*) CMC solution. This solution was inserted into 0.05 M citrate phosphate buffer (pH 4.0) and incubated at 45 °C for 30 min [15]. The enzymatic reaction was interrupted via the addition of 1.0 mL DNS reagent and by placing the solution into a boiling water bath for 10 min. A spectrophotometer measured the color of the reaction product as 540 nm. One enzyme unit was defined as the quantity of enzyme that hydrolyzed CMC to produce 1 μmol of sugar per minute under experimental conditions. The equation is derived from the glucose-equivalent factor generated in the assay in mmol of glucose, the volume of the enzyme being tested in the assay (0.5 mL), and the incubation time (30 min) required for the generation of the reducing equivalents [24].

Carboxymethyl cellulase activity (Unit/mL) = (C × D) ÷ MTV

Then: C = Releasing glucose from cellulase (mg)

D = Dilution factor of enzyme

µM = Glucose molecular weight (180 µg/µmol)

T = Time incubation

V = Enzyme volume

The purpose of this experiment was to select the appropriate media solution for the yeast to grow with the highest yield biomass and cellulase activity. Only one media so-lution was selected to expand the population of yeast before fermenting with RS in further experiments. The yeast was selected for three colonies, which are the highest biomass and cellulase producing colonies in the media solution. Therefore, the main consideration is to use the data from two parameters: biomass and cellulase production.

### 2.2. Study of Potential Ruminal Yeast and Identification

#### 2.2.1. Molecular Identification of Selected Ruminal Yeast

DNA isolation was performed by boiling lysis buffer cells according to Maniatis and Fritsch’s methods [20] with slight modifications. A loopful of yeast cells was transferred to a 1.5 mL Eppendorf tube and 100 µL of lysis buffer was added. The cell suspensions were boiled for 15 min in a water or metal block bath. After boiling, 100 μL of 2.5 M potassium acetate (pH 7.5) was added and placed on ice for 1 h, then centrifuged for 5 min at 14,000 rpm. Supernatant was extracted twice with chloroform:isoamyl alcohol (24:1 *v*/*v*). DNA was precipitated with isopropanol, placed on hold for 10 min at 20 °C, and centrifuged for 15 min at 15,000 rpm. DNA pellets were rinsed with 70% ethanol and 90% ethanol, then dried at room temperature for 15–30 min. The dried DNA was dissolved in 30 µL Milli-Q purified water.

The divergent D1/D2 domain of 26S rDNA was amplified with primers NL-1 (5′-GCA TAT CAA TAA GCG GAG GAA AAG-3′) and NL4 (5′-GGT CCG TGT TTC AAG ACG G-3′) [25]. Amplification was performed in 100 µL of reaction mixture conditioning 100 ng of 2.5 U of Taq polymerase, genomic DNA, 40 mM of each primer, 20 mM of each dNTP, 1.5 mM MgCl2, and 10 mM Tris-HCl. The reaction was pre-denatured at 94 °C for 5 min. This was repeated at 94 °C for 1 min for 30 PCR cycles, with annealing at 55 °C for 1 min, and extension at 72 °C for 2.5 min, followed by the final extension at 72 °C for 10 min. According to the manufacturer’s instructions, the amplified DNA was purified with a QIAquick PCR purification kit. Visualization of purified amplified DNA was accomplished by electrophoresis using 0.8% agarose gel in 1× tris-borate-EDTA (TBE) buffer and staining with ethidium bromide (8 × 10^−5^ μg/mL), observed under a UV illuminator.

The nucleotide sequences of the 26S rDNA D1/D2 domain were determined directly using PCR products according to Kurtzman and Robnett [25], with slight modifications. Cycle sequencing of the D1/D2 domain was carried out with the forward primer NL1 (5′-GCA TAT CAA TAA GCG GAG GAA AAG-3′) and reverse primer, NL4 (5′-GGT CCG TGT TTC AAG ACG G-3′), using an ABI PrismTM BigDye^TM^ Terminator Cycle Sequence Ready Reaction Kit (Applied Biosystems, Stafford, Texas, USA) according to the manufacturer’s instructions.

#### 2.2.2. Measurement of Yeast Cell Growth, Reducing Sugar and Ethanol Production

Potential yeasts were selected, identified, and analyzed in a 7 × 4 factorial use in a completely randomized design with three replications. Factor A was incubation time at 0, 12, 24, 36, 48, 60, and 72 h. Factor B was isolated yeast strains including codes H-KKU20 (as *P. kudriavzevii*-KKU20), I-KKU20 (*C. tropicalis*-KKU20), C-KKU20 (as *Galactomyces* sp.-KKU20), and S. cerevisiae. The optimized fermentation of the media was carried out following the appropriate solutions for potential yeast, biomass, and CMCase activity. The samples were collected in six 1.5 mL tubes with duplicates (two tubes each for cell count, reducing sugars, and ethanol production) for each incubation time at 0, 12, 24, 36, 48, 60, and 72 h. In the first two tubes, fresh samples were monitored immediately via the counting method using a hemocytometer under a microscope according to Darvishi et al. [26].

Another two tubes determined the reducing sugars using the 3,5-dinitrosalicylic acid (DNS) method [27]. A double-beam UV scanning spectrophotometer was used to measure absorbance. A reduction in sugar content before and after fermentation was determined by applying 1.0 mL of diluted solution (1 mL sample in 9 mL distilled water) to a test tube with 1.0 mL of DNS reagent. A blank was run in parallel with 1.0 mL of distilled water and 1.0 mL of DNS. The tubes were heated for 15 min in a bath of boiling water. Next, 5 mL of distilled water was applied after the tubes were cooled at room temperature, and absorbance values were noted at 540 nm. A reduction in the sugar concentration was determined from the standard glucose curve and via the dilution factors [28].

The last two tubes were centrifuged at 16,000 rpm for 15 min, and the liquid above the solid residue was filtered using a 0.45-micron syringe filter. Ethanol analyses were performed using an Agilent 7890B gas chromatograph (Agilent Corporation, Santa Clara, CA, USA). The standard water-based ethanol solutions were prepared from 0.0–1.0% (*v*/*v*), and 1–2 μL were injected into the gas chromatography (GC) injection port and then subjected to quantitative ethanol analysis using an HP-5 capillary column (length 30 m) on a GC apparatus (oven temperature 40 °C; constant flow mode with a Flame Ionization Detector (FID) 300 °C, air flow of 350 mL/min; inlet temperature of 150 °C) [29].

### 2.3. Statistical Analysis

#### 2.3.1. Dry Biomass and Carboxymethyl Cellulase Activity

Data for isolated yeast from rumen with different sugarcane molasses and urea concentrations were analyzed as a 12 × 3 × 3 factorial in a completely randomized design. The analysis of variance procedure of the SAS program was used for the analysis and the statistical model is as follows:

*Y_ijk_* = *μ* + *A_i_* + *B_j_* + *AB_ij_* + *C_k_* + *AC_ik_* + *BC_jk_* + *ABC_ijk_* + *ε_ijk_* (1)

where *Y_ijk_* = observation, *μ* = overall mean, *A_i_* = Yeast strain effect (i = a,b,c,d,e,f,g,h,i,j,k and *S. cerevisiae*), *B_j_* = sugarcane molasses effect (j = 5, 15 and 25%), *AB_ij_* = yeast strain effect × sugarcane molasses effect, *C_k_* = urea effect (k = 1, 3 and 5%), *AC_ik_* = yeast strain effect × urea effect, *BC_jk_*_=_ sugarcane molasses effect × urea effect, *ABC_ijk_* = yeast strain effect × sugarcane molasses × urea effect and *ε_ijk_* = error.

#### 2.3.2. Cell Counts, Ethanol Production and Reducing Sugar

Data for isolated yeast strains and incubation time were analyzed as a 7 × 4 factorial in a completely randomized design. The ANOVA procedure of the SAS program was used for the analysis and the statistical model is as follows:

*Y_ij_* = *μ* + *A_i_* + *B_j_* + *AB_ij_* (2)

where *Y_ijk_* = observation, *μ* = overall mean, *A_i_* = Incubation time effect (i = 0, 12, 24, 36, 48, 60 and 72 h), *B_j_* = different yeast strains effect (j = H-KKU20 (as *P. kudriavzevii*-KKU20), I-KKU20 (as *C. tropicalis*-KKU20), C-KKU20 (as *Galactomyces* sp.-KKU20) and *S. cerevisiae*, *AB_ij_* = incubation time effect × isolated yeast strains effect and *ε_ijk_* = error.

Treatment means were calculated using the Least Square Means (LSMEANS) option of SAS The entire experimental design used ANOVA and the General Linear Model (GLM) procedures of SAS [30] (Version 6.0; SAS Institute Inc., Cary, NC, USA). When F-tests were significant, single-degree-of-freedom orthogonal polynomials were used to determine the trends of factors. The mean treatment differences were determined by Duncan’s New Multiple Range Test (DMRT) at *p* = 0.05 [31].

## 3. Results

### 3.1. Isolation and Morphological Characteristics of Yeast Isolated from Rumen Fluids

Isolation was imposed under aerobic conditions resulting in 11 different colonies whose morphological and microscopic observations are shown in Table 1. The 11 isolates of yeast were grown on YM agar plates and selected for their formed appearance, elevation, colony nature, and colony color. The budding stage of the isolated yeast was observed under a 40× microscope; the colonies were confirmed to be yeast as shown in Table 1. Two appearances of colonies were noted including asymmetric colonies of isolated yeast (indicated as A, B, C, E, and J) and ovoid colonies (coded as D, F, G, H, I, and K). In addition, elevations of colonies are indicated as A and B whereas flat colonies are coded as C, D, E, I, and J; convex colonies are indicated as F, G, H, and K. Smooth colonies are coded as D, E, I, and J while other codes correspond to rough colonies. Most colonies in this study were white except codes D and J, which were colorless. Code G was turbid.

### 3.2. The Biomass Production and Cellulase Activity

#### 3.2.1. Effects of Varying Concentrations of Sugarcane Molasses and Urea on Biomass of Isolated Yeast at 72 h of Incubation Time

**Figure 1:**
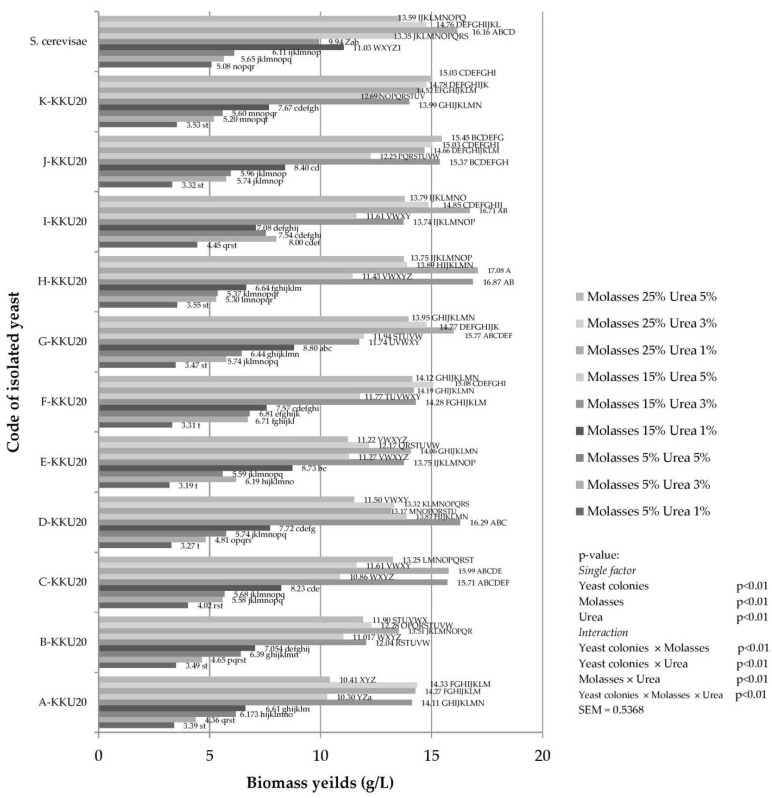
Biomass (horizontal axis) for yeast strains grown in different sugarcane molasses (5, 15 and 25%) with urea (1, 3 and 5%) for 72 h. ^A–Z,a–t^ Means with different superscript letters within bar chart are considered statistically significant (*p* < 0.01), SEM = standard error of the mean, *S. cerevisiae* = *Saccharomyce cerevisiae*.

Interactions were observed between yeast strains and sugarcane molasses with urea on biomass production (Figure 1). Dry biomass production from ruminal isolated yeast was observed from 3.19 to 17.08 g/L in all media solutions.

Isolated yeast from the rumen could provide a high amount of biomass relative to the other media solution (*p* < 0.01) when the inoculant consisted of M15 + U3, M25 + U1, M25 + U3, and M25 + U5. In addition, 11 isolates of biomass-producing yeasts were found with a media solution of M25 + U1 whereas M15 + U3, M25 + U3, and M25 + U5 were found by 8, 7, and 2 isolates, respectively.

#### 3.2.2. Effects of Varying Concentrations of Sugarcane Molasses and Urea on Cellulase Activity of Isolated Yeast at 72 h of Incubation Time

**Figure 2:**
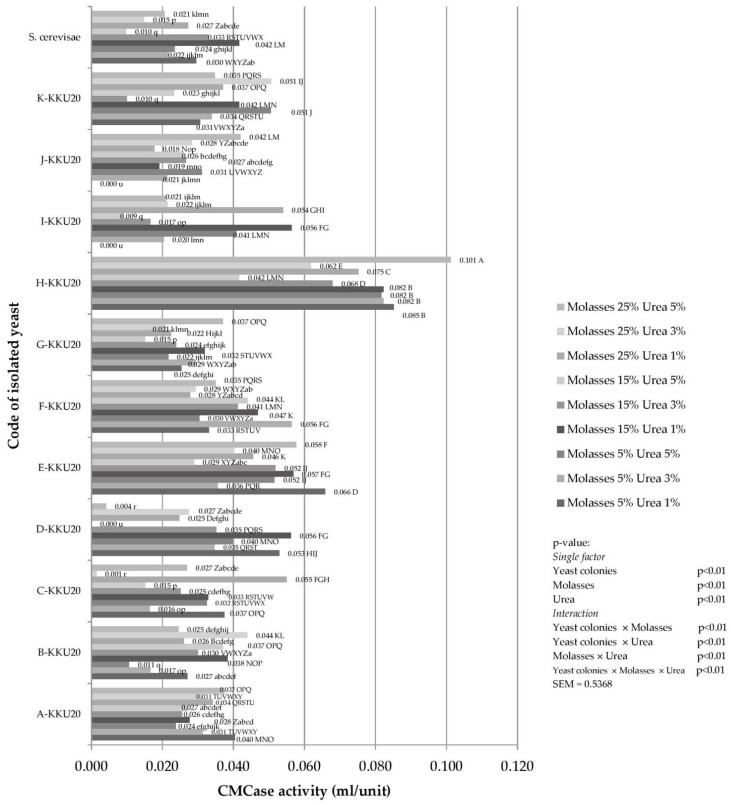
Effect of CMC concentration from 1% (*w*/*v*) on cellulase production of each rumen fluids isolates yeast at 72 h. ^A–Z,a–u^ Means with different superscript letters within a bar chart are considered statistically significant (*p* < 0.01), SEM = standard error of the mean, *S. cerevisiae* = *Saccharomyce cerevisiae*.

Interactions were observed between yeast strains and sugarcane molasses with urea in terms of carboxymethyl cellulase activity (Figure 2). Isolated yeast from the rumen could provide a high amount of CMCase when inoculated in all media solutions except M15 + U5 (*p* < 0.01). There were four isolates of cellulase-producing yeasts discovered in the media solution of M25 + U1 and M25 + U5, whereas M5 + U1, M5 + U3, M5 + U5, M15 + U1, M5 + U3, and M25 + U3 were found with 2, 3, 1, 2, 1, and 2 isolates, respectively.

### 3.3. Selection and Identification of Potential Yeast Strains

Based on these criteria, only one media solution was selected to expand the population of the yeast before fermenting with RS. We selected M25 + U1 because it has the best potential and expands more than other media solution groups. The top three isolated yeasts from the media solution of M25 + U1 include H-KKU20, I-KKU20, and C-KKU20; these were chosen after considering the CMCase activity. M25 + U1 is a media solution with the same potential for yeast release of CMCase as M25 + U5. However, consideration along with biomass production makes M25 + U1 stand out. It is better than M25 + U5 because it requires a low N source to produce CMCase. Furthermore, H-KKU20 does not provide the best CMCase relative to M25 + U1 group, but it is the highest. Therefore, these yeast isolates (H-, I-, and C-KKU20) were used for further evaluation.

The newly isolated yeast strain was identified via DNA sequencing [25] using the 26S rRNA gene D1/D2 domain. Identification of isolates H-KKU20 and I-KKU20 revealed that those isolates belonged to *Pichia kudriavzevii*-KKU20 and *Candida tropicalis*-KKU20. The D1/D2 sequence of C-KKU20 had 99.82% (1 nucleotide substitution) similarity with the undescribed species *Galactomyces* sp. HN21-4 (EU651849) (name changes: *Geotrichum* sp. HN21-4) and was closest to the *Galactomyces geotrichum* strain NRRL Y-17569T (NG_054826) but with 11 nucleotide substitutions and one gap. Strain C-KKU20 was identified as *Galactomyces* sp.-KKU20 (Table 2) based on the sequence of the D1/D2 region.

### 3.4. Cell Counts, Ethanol Production and Reducing Sugar by Ruminal Yeast Strains

#### 3.4.1. Effect of Incubation Time and Isolated Yeast Strains on Cell Counts

**Figure 3:**
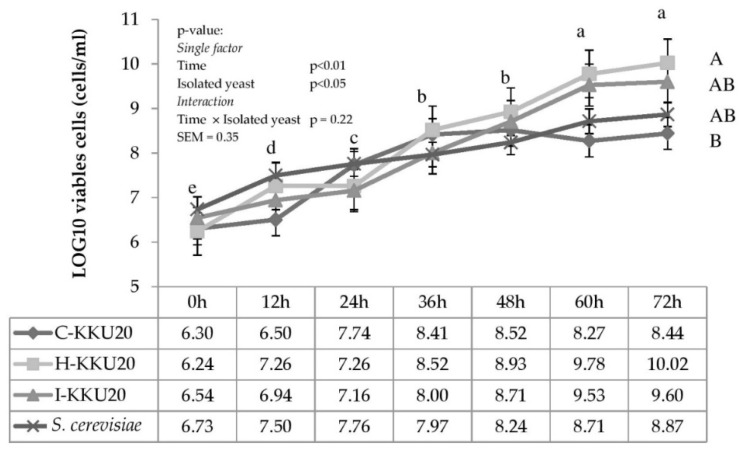
Viable cell count from batch fermentation under sugarcane molasses 25% with urea 1% plus 1% CMCase in the incubator shaker at 30 °C and 150 rpm for 72 h. ^A,B^ Means with different superscript letters for the effect of yeast colonies are significant at *p* < 0.05, ^a–e^ Means with different superscript letters for the effect of time are significant at *p* < 0.01. SEM = standard error of the mean, *S. cerevisiae* = *Saccharomyce cerevisiae*.

The influences of various times and yeast strains on cell count are illustrated in Figure 3. There were no interactions between time and yeast in terms of cell count (*p* > 0.05). Yeast cells were counted from 6.24 to 10.02 Log cells/mL from over 0 to 72 h of incubation time. *P. kudriavzevii*-KKU20 provided the maximum cell growth at 10.02 Log cell/mL at 72 h, whereas *S. cerevisiae* showed the lowest cell growth at 8.87 Log cell/mL at 72 h (*p* < 0.05). Yeast-viable cells increased along with incubation time. Maximum cell growth occurred at 60 and 72 h after incubation (*p* < 0.01). It is possible to proliferate the yeast count cycle in just 60 h.

#### 3.4.2. Effect of Incubation Time and Isolated Yeast Strains on Ethanol Production

**Figure 4:**
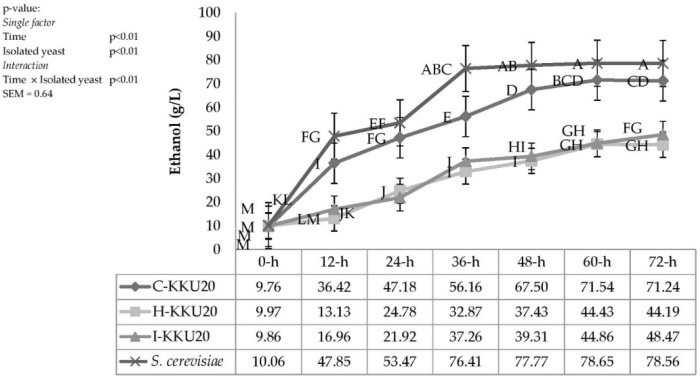
The production of ethanol by four different yeast strains under sugarcane molasses 25% with urea 1% plus 1% CMC in the incubator shaker at 30 °C and 150 rpm for 72 h. ^A–M^ Means with different superscript letters for the interaction effect of time × yeast colonies are significant at *p* < 0.01. SEM = standard error of the mean, *S. cerevisiae* = *Saccharomyce cerevisiae*.

*Galactomyces* sp.-KKU20, *C. tropicalis*-KKU20, *S. cerevisiae*, and *P. kudriavzevii*-KKU20 were evaluated for ethanol production, as shown in Figure 4. Interactions were observed between incubation time and isolated yeast strains (*p* < 0.01). The isolated yeast strains produced about 9.76 to 78.6 g/L ethanol at 0 to 72 h of incubation time. The largest amount of ethanol production was observed in *S. cerevisiae*: 76.4, 77.8, 78.5, and 78.6 g/L at 36, 48, 60, and 72 h of incubation time, respectively (*p* < 0.01). *P. kudriavzevii*-KKU20 and *C. tropicalis* -KKU20 produced the least amount of ethanol: 32.9, 37.4, 44.4, and 44.2 g/L and 37.3, 39.3, 44.9, and 48.5 g/L at 36, 48, 60, and 72 h of incubation time, respectively (*p* < 0.01).

#### 3.4.3. Effect of Incubation Time and Isolated Yeast Strains on Reducing Sugar

**Figure 5:**
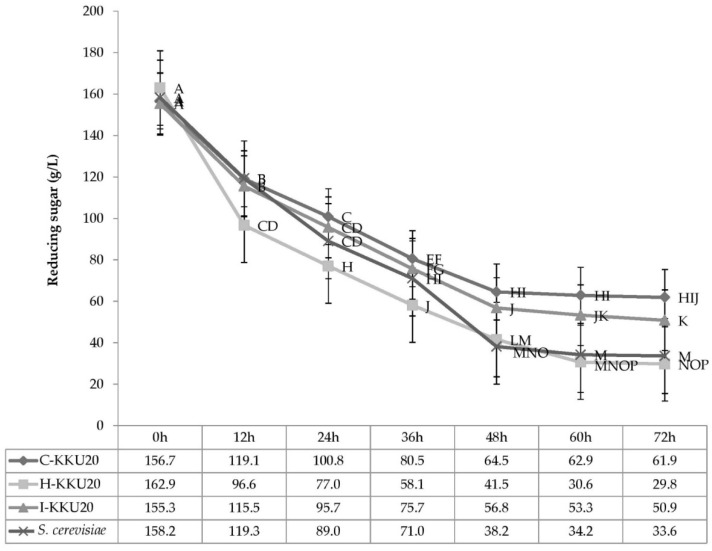
Sugar use by four different yeast strains under sugarcane molasses 25% with urea 1% plus 1% CMC in the incubator shaker at 30 °C and 150 rpm for 72 h. ^A–P^ Means with different superscript letters for the interaction effect of time × yeast colonies are significant at *p* < 0.01. SEM = standard error of the mean, *S. cerevisiae* = *Saccharomyce cerevisiae*.

Interaction effects were observed between incubation time and isolated yeast strains (*p* > 0.05) in terms of reducing sugar (Figure 5). The isolated yeast yielded reduction of sugars by between 162.9 and 29.8 g/L from 0 to 72 h of incubation time. *P. kudriavzevii*-KKU20 yielded the lowest amount of reduction in sugar, at about 30.6 and 29.8 g/L at 60 and 72 h of incubation time, respectively. Meanwhile, the two strains that yielded the greatest reduction in sugar were *Galactomyces* sp.-KKU20 and *S. cerevisiae* at 60 and 72 h of incubation time, respectively.

## 4. Discussion

### 4.1. Isolation of Yeast from Rumen Fluids

Yeast isolates were ovoid (6 in 11), flat (5 in 11), convex (4 in 11), or rough (7 in 11). White colonies (9 in 11) appeared most frequently. Yeast was detected similarly to Marrero et al. [32], who concluded that the yeast morphologies in their study were slightly convex, smooth, and white- to cream-colored, which is typical of ruminal yeast. However, many factors might cause different types of yeast to be discovered such as feed sources, roughage per concentrate ratios (R:C), and ruminant species. Marrero et al. [33] isolated yeast from dairy cattle and found that some isolated yeasts could present a pink coloration, which was later discovered to be *Levica* strains 18 (L18). Thus, the morphological characteristics of ruminal yeast colonies can be quite complex.

### 4.2. The Biomass Production and Cellulase Enzyme Activity of Isolated Yeasts

To increase the biomass of yeast, a substrate such as soluble carbohydrate and nitrogen must provide sufficient supplies for the growth of yeast cells [34]. Paserakung et al. [35] reported that increasing the molasses concentration from 8% to 16% resulted in the greatest biomass production of 25.9% as obtained from *Trichosporon asahii*. In addition, Johnson, Singh, Saini, Adhikari, Sista, and Yadav [23] studied the effects of different single-substrate carbon sources such as molasses, glucose, and sucrose with limited nitrogen sources in media solutions on the biomass production of *Rhodotorula glutinis* IIP-30. They found that biomass production increased by 87.8% in the molasses treatment. Thus, molasses might be a better potential carbon source for *Trichosporon asahii* growth than other carbon sources [6]. Our media solution contained 25% molasses with 1% urea providing a maximum ruminal yeast biomass of 29.2%. This signifies that providing optimum levels of molasses and urea could positively affect biomass production. However, only molasses was used as carbon source in these experiments; thus, another carbon source should be elucidated.

Manikandan and Viruthagiri [36] reported that the nitrogen source, concentration of nitrogen, and carbon/nitrogen ratio (C:N ratio) also influenced the production of biomass. Danesi et al. [37] demonstrated that the use of sugarcane blackstrap molasses and yeast extract at a C:N ratio of 10:1 provided the greatest biomass from the yeast. In addition, Sokchea et al. [38] found that the biomass of yeast was highest at 7.57 g/L when the C:N ratio reached 10:1. Compared to our study, however, the C:N ratio was 25:1, and this high carbon source might lead to a higher level of biomass production (17.07 g/L). Proper proportions of carbon and nitrogen can increase yeast growth as in previous experiments where live yeast inoculant was introduced into molasses and urea could increase yeast count and increase animal growth. Boonnop, Wanapat, Nontaso, and Wanapat [6] reported that protein increased from 3.4% to 32.5% in cassava chips (93.5% is true protein) after the yeast proliferated; lysine increased from 3.8% to 8.5%. While experimenting with cassava root, Khampa, Chuelong, Kosonkittiumporn and Khejornsart [7] found that the effect of yeast fermentation increased the amount of protein in cassava chips by up to 36.1%. However, Khampa et al. [39] showed that the resulting protein component in cassava chips only increased by 19.2%.

Here, the isolated yeast produced and released cellulase enzymes ranging from 0.020 to 0.075 units/mL. This is the first time that this result has been achieved, which might support the breaking down of fiber content in the rumen. Although studies on the release of cellulase enzymes by yeast from the rumen have not yet been conducted, isolated yeasts from natural sources have demonstrated that yeast could produce cellulase enzymes. Sarawan [22] revealed that *Candida glabrata*, *Candida natalensis*, and *Kluyveromyces africanus* (isolated the from *Jasminum adenophyllum* plant) can release cellulase enzymes ranging from 0.004 to 0.08 units/mL when cultured in yeast extract peptone dextrose broth with 1% CMC. Thus, cellulase enzymes produced by yeasts might be a potential digest feed due to their cellulose content. Mechanistically, cellulose is hydrolyzed by cellulase as it breaks down the β-1,4-glycosidic bonds [40]. Cellobiohydrolases (CBHs, EC 3.2.1.91) are important cellulase enzymes found in yeast and are instrumental in high-performance natural cellulose hydrolysis [41]. Examples of CBH expressed in yeasts include CBH1 (Cel7A) and CBH2 (Cel6A) [42].

### 4.3. Identification of Isolated Yeasts

Our study isolated three yeasts from rumen fluids: *P. kudriavzevii*-KKU20, *C. tropicalis*-KKU20, and *Galactomyces* sp.-KKU20. The name KKU refers to Khon Kaen University where the strain was originally isolated, and the number “20” means the year of discovery, 2020. Interestingly, similar yeast species (*P. kudriavzevii*, *C. tropicalis*, and *Galactomyces* sp.) have previously been isolated [43,44,45,46]. However, the qualities of these strains have not been studied, and this is the first report on their characteristics such as biomass production, cellulase activity, growth patterns, ethanol production, and the reducing sugars of the yeast from rumen. In addition, the types of potential yeast were Crabtree-negative. Crabtree-negative yeasts *P. kudriavzevii*-KKU20 and *C. tropicalis*-KKU20 can more rapidly convert available sources of carbon into a solution for yeast biomass. This trend led to consideration of ruminant nutrition as a candidate for the development of protein sources in feed.

### 4.4. Cell Counts, Ethanol Production and Sugar Reduction of Selected Ruminal Yeasts

Our results showed that the *Pichia kudriavzevii*-KKU20 inoculants in media solution show increased growth versus other species. The maximum yeast growth was 10.02 Log cells/mL in aerobic conditions at 72 h of incubation. *Pichia kudriavzevii*-KKU20 was classified as a Crabtree-negative group; the ability to propagate cells is greater than for the Crabtree-positive group because the glucose use is high. Crabtree-negative yeasts can transport glucose by an inducible high-affinity proton symport mechanism. Aerobic conditions provide especially steady growth conditions [47].

These results clearly indicate that *S. cerevisiae* can produce alcohol when used as an inoculant with a high concentration of carbon under aerobic conditions. Under aerobic conditions, *S. cerevisiae* produced 78.6 g/L of ethanol after 72 h, which was more than other species produced. The high ethanol production capability of *S. cerevisiae* occurs because the pyruvate dehydrogenase complex enzyme is inhibited when its inoculant in media solution contained a large amount of sugar [48]; pyruvate decarboxylase is activated instead (three- to four-fold) and changes sugar to ethanol (although there is sufficient oxygen) [49]. Here, the high level of molasses (25% under aerobic conditions) could also have allowed *S. cerevisiae* to produce a higher ethanol concentration versus other yeast species.

The sugar consumption by yeasts is related to their growth curve and depends on the yeast species [50]. Isolated yeast strains from rumen have a higher growth curve than Crabtree-positive yeasts such as *S. cerevisiae.* This experiment revealed that *P. kudriavzevii*-KKU20 consumed more sugar (133.1 g) from 0 to 72 h of incubation than *S. cerevisiae*, which consumed only 124.6 g of sugar. Van Urk et al. [51] stated that the sugar consumption rate of a pyruvate decarboxylase-deficient mutant of *S. cerevisiae* is much lower than that of Crabtree-negative yeast strains, indicating that pyruvate decarboxylase could have a strong influence on the glycolytic flux.

Different yeast species might be involved in biomass production. Van Urk, Voll, Scheffers, and Van Dijken [51] reported that *S. cerevisiae* had low proliferation potential when excessive glucose was present even under aerobic conditions. Wardrop, Liti, Cardinali, and Walker [14] found that *Kluyveromyces marxianus* provided a larger amount of biomass (seven times greater) than *S. cerevisiae* when cultured in a media solution with excessive glucose. Oxygen acts as the final electron acceptor under aerobic conditions. If the yeasts possess a complete metabolism (e.g., *P. kudriavzevii*-KKU20 and *C. tropicalis*-KKU20), then they will produce a large amount of biomass and less alcohol—this condition is called a “Crabtree-negative yeast”. In contrast, *S. cerevisiae* exhibits alcoholic fermentation and produces high amounts of ethanol, i.e., “Crabtree-positive yeast” [48]. This explains why *P. kudriavzevii*-KKU20 and *C. tropicalis*-KKU20 produced a larger amount of biomass (more than *S. cerevisiae*).

The real potential of yeasts as protein sources in ruminant feeding lies in the fact that isolated yeast could increase the amount of biomass produced versus the 11 yeasts and *S. cerevisiae.* The biomass and cell count data obtained here provide us with an opportunity to create feed for animals from yeast cells that is rich in proteins, essential amino acids, and vitamins. Therefore, the use of different yeast strains may produce interesting results. In addition, cellulase activity increases the possibility of the use of yeast in roughage such as RS, which may add nutritional value.

## 5. Conclusions

Screening and isolating yeasts from rumen fluids resulted in 11 different yeasts. The most valuable yeasts discovered in the rumen fluid of cattle were *Pichia kudriavzevii*-KKU20, *Candida tropicalis*-KKU20, and *Galactomyces* sp.-KKU20, which produce a large amount of biomass and cellulase. The maximum growth of isolated yeast was shown in media containing 25% sugarcane molasses and 1% urea with pH 3.5 and 150 rpm shaking. Under these circumstances, *P. kudriavzevii*-KKU20 has great potential in terms of biomass production, cellulase enzyme activity, and cell number. However, an evaluation of *Pichia kudriavzevii*-KKU20’s ability in terms of fiber improvement and yeast biomass production needs to be carried out to assess the increased nutritive value for ruminant animals.

## Figures and Tables

**Table 1:** Colonies morphology of ruminal yeast strains of Thai-Holstein-Friesian.

Code Name	Picture	Appearance	Elevation	Colony Nature	Colony Color
A-KKU20	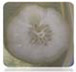	Asymmetrical	Raised	Rough	White
B-KKU20	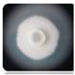	Asymmetrical	Raised	Rough	White
C-KKU20	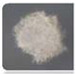	Asymmetrical	Flat	Rough	White
D-KKU20	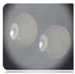	Ovoid	Flat	Smooth and shiny	colorless
E-KKU20	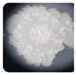	Asymmetrical	Flat	Rough	White
F-KKU20	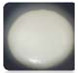	Ovoid	Convex	Smooth	White
G-KKU20	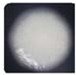	Ovoid	Convex	Rough	Turbid
H-KKU20	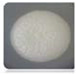	Ovoid	Convex	Rough	White
I-KKU20	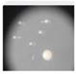	Ovoid	Flat	Smooth	White
J-KKU20	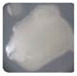	Asymetrical	Flat	Smooth	Colorless
K-KKU20	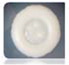	Ovoid	Convex	Rough	White

**Table 2:** Identification of isolated yeast from rumen fluids.

Isolates	Gene Bank Accession No.	Nearest Species with Accession No.	Nucleotide Identity (%)	No. of Nucleotide Differences
H-KKU20	MH545928	*Pichia kudriavzevii*	572/572 (100)	0
I-KKU20	U45749	*Candida tropicalis*	570/570 100	0
C-KKU20	EU651849	*Galactomyce* spp.	552/553 (99.82)	1

## Data Availability

Data available in a publicly accessible repository.
